# Supplementary material for: EnzML: multi-label prediction of enzyme classes using InterPro signatures
Source: BMC Bioinformatics. 2012 Apr 25;13:61. doi: 10.1186/1471-2105-13-61 (PMC3483700; doi:10.1186/1471-2105-13-61)
Supplement: Addtional file 5 — The Java code to format the data files, evaluate and predict. The file enzml_java_code.tar.gz contains the Java code used to format database data to ARFF and XML formats, to execute cross and train-test (jackknife) evaluations and to record evaluation results to database. More information is included in the readme.txt file and the Javadoc files. The code can be used with a MySQL database. To use a different database software, other JDBC drivers might be required. [file 1471-2105-13-61-S5.gz › java_code/utils/doc/edu/cornell/lassp/houle/RngPack/RandomJava.html]

RandomJava


---


|  |  |  |
| --- | --- | --- |
| **Overview** \| **Package** \| **Class** \| **Use** \| **Tree** \| **Deprecated** \| **Index** \| **Help** | |  |
| **PREV CLASS**   **NEXT CLASS** | **FRAMES**    **NO FRAMES**     **All Classes** |  |
| SUMMARY: NESTED \| FIELD \| CONSTR \| METHOD | DETAIL: FIELD \| CONSTR \| METHOD |  |


---


## edu.cornell.lassp.houle.RngPack Class RandomJava

```
java.lang.Object
  cern.colt.PersistentObject
      edu.cornell.lassp.houle.RngPack.RandomElement
          edu.cornell.lassp.houle.RngPack.RandomJava
```

**All Implemented Interfaces:**: java.io.Serializable, java.lang.Cloneable

---

``` public class RandomJava extends RandomElement ```

RandomJava is a class wrapper for the `Math.random()` generator
that comes with Java. I know nothing about the quality of
`Math.random()`, but I will warn the reader that system-supplied
RNGs have a bad reputation; RandomJava is **NOT** reccomended for
general use, it has only been included as a straightforward example of how to
build a `RandomElement` wrapper for an existing RNG. The
RANMAR, RANECU and RANLUX generators included in
this package all appear to be faster than Math.random(); all three are
well-studied, portable and proven in use.

Source code  is available.

**Version:**
:   1.0

**Author:**
:   Paul Houle  (E-mail: houle@msc.cornell.edu)

**See Also:**: `Ranmar`, `Ranlux`, `Ranecu`, Serialized Form

---

**Constructor Summary**

`RandomJava()`


| **Method Summary** | |
| --- | --- |
| `double` | `raw()`             Wrapper for `Math.random().` |

**Methods inherited from class edu.cornell.lassp.houle.RngPack.RandomElement**

`choose, choose, gaussian, gaussian, powlaw, raw, raw, uniform`

**Methods inherited from class cern.colt.PersistentObject**

`clone`

**Methods inherited from class java.lang.Object**

`equals, getClass, hashCode, notify, notifyAll, toString, wait, wait, wait`

**Constructor Detail**

### RandomJava

```
public RandomJava()
```


**Method Detail**

### raw

```
public double raw()
```

:   Wrapper for `Math.random().`

    :   **Specified by:**: `raw` in class `RandomElement`
    :   **Returns:**: a random double in the range (0,1). **See Also:**: `RandomElement.raw()`


---


|  |  |  |
| --- | --- | --- |
| **Overview** \| **Package** \| **Class** \| **Use** \| **Tree** \| **Deprecated** \| **Index** \| **Help** | |  |
| **PREV CLASS**   **NEXT CLASS** | **FRAMES**    **NO FRAMES**     **All Classes** |  |
| SUMMARY: NESTED \| FIELD \| CONSTR \| METHOD | DETAIL: FIELD \| CONSTR \| METHOD |  |


---
